# Evaluation of clotting factor activities early after severe multiple trauma and their correlation with coagulation tests and clinical data

**DOI:** 10.1186/s13017-015-0038-1

**Published:** 2015-09-22

**Authors:** Manuel Burggraf, Arzu Payas, Max Daniel Kauther, Carsten Schoeneberg, Sven Lendemans

**Affiliations:** Department for Orthopaedics and Emergency Surgery, University Hospital Essen, University Duisburg-Essen, Hufelandstr. 55, 45147 Essen, Germany; Clinic for Accident Surgery and Orthopaedics, Alfried Krupp Hospital Steele, Hellweg 100, 45276 Essen, Germany

**Keywords:** Severe multiple trauma, Injury, Coagulation, Coagulopathy, Clotting factor, Coagulation factor, International normalized ratio, Partial thromboplastin time

## Abstract

**Introduction:**

Traumatic injuries are amongst the leading causes of death worldwide, frequently as a result of uncontrolled hemorrhage. Critical deficiencies in clotting factors have been noted in trauma-induced coagulopathy. However, the exact underlying conditions that result in devastating coagulopathies remain unclear. The purpose of this study was to elucidate these underlying deficiencies.

**Methods:**

Blood samples were drawn from 45 severely injured trauma patients on their arrival at the resuscitation room, and the activities of all soluble clotting factors and routine coagulation tests were assessed. The Mann–Whitney-*U*-test was used to assess differences in coagulation activity between the patients and healthy controls. Furthermore, Spearman’s rank correlation was used to analyze the blood work.

**Results:**

After severe trauma the levels of serum fibrinogen and calcium were significantly reduced. Furthermore, traumatized patients had a significantly increased International Normalized Ratio (INR) compared to healthy controls. The median activities of all clotting factors were reduced after severe multiple trauma, with the exception of factor VIII, which was increased. Statistically significant differences were observed for factors II (80 vs. 122 %, *P* < 0.0001), V (76 vs. 123 %, *P* < 0.0001), VII (90 vs. 114 %, *P* = 0.002), VIII (200 vs. 108 %, *P* < 0.0001), and X (86 vs. 122 %, *P* < 0.0001). Spearman’s correlation indicated a significant negative correlation between INR on arrival with fibrinogen and levels of factors II, V, and VII, whereas Partial Thromboplastin Time was significantly negatively correlated with factor VIII (all *P* < 0.0001).

**Conclusions:**

These findings suggest a general but rather moderate impairment of clotting factor activities following severe multiple trauma. In the concept of a calculated coagulation therapy, this could demand for the use of factor concentrates with higher ratios of clotting factors. Finally, the physiological importance of strongly elevated factor VIII activity remains unclear, but a possible interference with *ex vivo* measurements of Partial Thromboplastin Time has to be considered.

## Introduction

Traumatic injuries account for more than 5 million deaths annually and are among the leading causes of death worldwide [1]. As many as 40 % of all mortalities after severe multiple trauma are related to uncontrolled hemorrhage [2, 3]. Several studies have reported a high incidence of coagulopathy on admission to the emergency department, and in this patient subgroup, mortality is increased by up to 4-fold compared to patients without coagulation abnormalities [4–8]. In fact, hemorrhage is the most common preventable cause of death after severe trauma and might be controlled by early aggressive therapy, either by surgery or correction of coagulopathy [9].

Brohi et al. demonstrated that systemic hypoperfusion in conjunction with tissue damage leads to widespread activation of activated protein C and liberation of tissue plasminogen activator, potentially causing systemic anticoagulation and hyperfibrinolysis [10, 11]. Hypothermia and acidemia further provoke the development of these clotting disorders [12–14]. Additionally, dilution and consumption of clotting factors may also significantly worsen bleeding disorders in major trauma patients [7]. However, little is known about the actual underlying clotting factor activities after severe injury. Early studies have generally focused on post-transfusional dysfunction at a later phase of the disorder and have described only a small subset of coagulation factors [15–18]. Recent studies from Rizoli et al. and Cohen et al. investigated a broader panel of clotting factors and indeed reported a significant correlation between clotting factor deficiencies and transfusion requirements [19, 20]. However, inclusion criteria aimed at coagulopathic subgroups of patients, either by selecting patients with highly reduced clotting factor activity (<30 %) or those with demonstrated transfusion requirements (at least one unit of red blood cells). It therefore remains unclear to what extent clotting factor deficiencies may generally exist in multiple trauma patients irrespective of the further course.

For this study we investigated the potential derangements of all soluble clotting factor activities early after serious injury and discuss if these changes could be estimated by standard coagulation tests.

## Methods

### Patients and normal donors

Adult patients were screened for enrollment if they were admitted directly from the scene of an accident to the trauma resuscitation room of our institution (Level 1 academic trauma center in Germany). Only severely injured patients with an Injury Severity Score (ISS) of at least 16 points were included. The study was performed in accordance with the Declaration of Helsinki and approved by the relevant local ethics committee (reference 12-5120-BO). Participants provided written informed consent. Pregnant women and patients known to have congenital coagulopathies or who were on anticoagulant medications were excluded. Ten healthy adult donors served as the control group.

### Blood samples

Directly after admission to the resuscitation room, blood samples were drawn from the femoral artery. In addition to routinely collected blood samples, an additional citrate syringe was obtained to assess clotting factor activity. In healthy controls, blood was drawn from a cubital vein. Immediately after collection, blood samples were transferred to the hospital laboratory, and standard coagulation tests for International Normalized Ratio (INR), Partial Thromboplastin Time (PTT), fibrinogen, and calcium were performed. In addition, the activities of clotting factors II, V, VII, VIII, IX, X, XI, XII, and XIII were analyzed. Analysis was performed by comparing samples to standard human plasma assays of clotting factors (SHP, Dade Behring Marburg GmbH, Marburg, Germany). The results were expressed as a percentage of standard activity. If immediate testing of clotting factor activity was not possible, e.g., during off-duty hours, specimens were cryo-stored at −70 °C until the final analysis could be performed the following weekday. Storage of frozen plasma samples is widely accepted and has not been specifically shown to interfere with clotting factor activity [21].

### Statistical analysis

Demographic data is reported as mean and standard deviation (SD) when applicable, while results are reported as median values. Differences between demographic data for the study groups were analyzed by *t*-test (age) or Fisher’s exact test (gender). Differences in clotting factor activity among patients and healthy controls were tested using the Mann–Whitney-*U*-test. Differences in INR, PTT, serum fibrinogen, and calcium among study groups were tested in the same manner. In a second step, Spearman’s rank correlation coefficient, *rho* (*ρ*), was calculated for traumatized patients by analyzing the results of routine coagulation tests as well as clinical data and clotting factor activity assessments. The 95 % confidence interval (CI) was computed by bootstrapping using a *bias-corrected and accelerated method* based on 1000 bootstrap samples. The correlation was considered negligible for absolute values of *ρ* between 0.0 and 0.2, weak between 0.21 and 0.4, moderate between 0.41 and 0.7, strong between 0.71 and 0.9, and very strong between 0.91 and 1. A *P* value smaller than 0.05 (2-tailed) was considered statistically significant for all tests. Data analysis was strictly exploratory. There was no correction for multiple testing. Data were analyzed and graphs were produced using IBM® SPSS® Statistics, Version 20 (Release 20.0.0).

All authors had access to primary clinical data.

## Results

### Demographic data

A total of 92 patients were enrolled in this study. A total of 45 (49 %) fully met the inclusion criteria for analysis and were included for testing against the healthy controls. Of the enrolled patients, 23 (25 %) were excluded from the study because the final evaluation revealed an ISS below 16, 11 (12 %) were too young, and 6 (6 %) were transferred from other hospitals. Three patients were on anticoagulant medication, and three had no history of trauma. One patient died within minutes of their admission. The baseline characteristics of the study groups are summarized in Table 1. The mean age of patients and healthy controls differed slightly but not significantly (46 years in the patient group and 40 years in the control group, *P* = 0.165). Overall, 80 % of patients and 70 % of healthy controls were male (*P* = 0.673). On average, patients were admitted to our hospital 66 ± 24 min after trauma. Further clinical data is given in Table 2 for the patient group.

**Table 1:** Demographic characteristics of the study population

Characteristics	Patients (*n* = 45)	Healthy controls (*n* = 10)	*P*
Age; years ± SD	46 ± 19	40 ± 9	0.165
Gender; male (%)	36 (80 %)	7 (70 %)	0.673
ISS; points ± SD	31 ± 9	n/a	n/a
Mechanism; blunt (%)	43 (96 %)	n/a	n/a
Mortality; n (%)	12 (27 %)	n/a	n/a

*n/a* not applicable

**Table 2:** Clinical data of the patient group

SBP (prehospital)	SBP (admission)	Lactate	Base excess	Temperature	Hemoglobin	Thrombocytes
124 (±37) mmHg	128 (±25) mmHg	2.4 (±2.0) mmol/l	−3.1 (±4.4) mmol/l	35.7 (±1.1) °C	12.0 (±2.3) g/dl	202 (±55) / nl

Data reported as means (± standard deviation)

*SBP* systolic blood pressure, *C* celsius

### Routine tests of coagulation

**Fig. 1:**
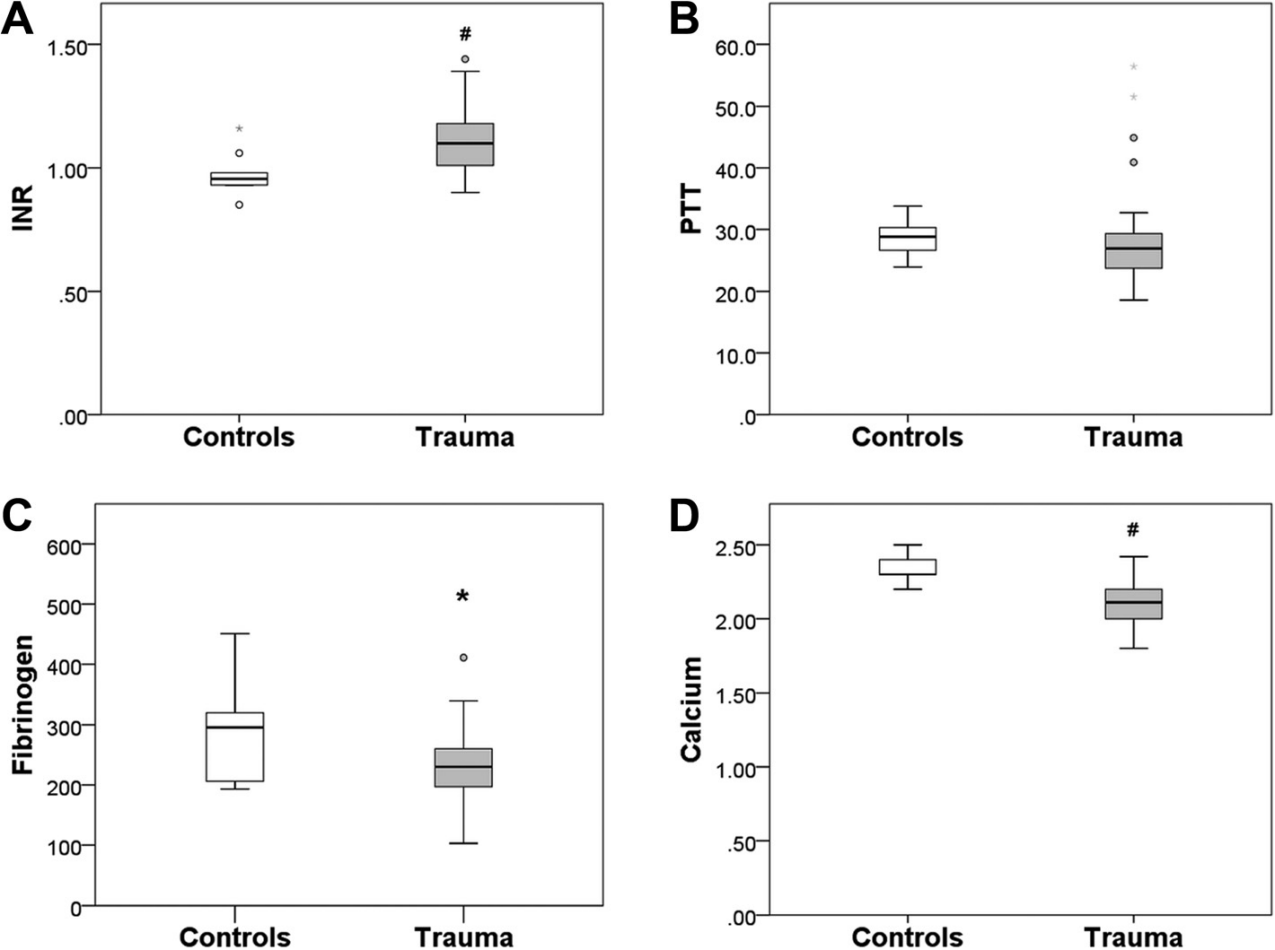
Results of routine coagulation tests. The results are presented as boxplots, bottom and top of the box indicate the 25th and 75th percentile or interquartile range (IR). The horizontal bar within the box represents the median. Whiskers indicate spread (1.5 times IR). Outliers (1.5 to 3 times IR) are indicated by circles, while extremes (greater than 3 times IR) are indicated by stars. The ordinate shows (**A**) INR ratio; (**B**) PTT seconds; (**C**) fibrinogen mg/dL; (**D**) calcium millimole per liter. *, *P* < 0.05; ^#^, *P* < 0.0001

Figure 1 shows the results of the routine coagulation tests. On admission to the resuscitation room, the median levels of serum fibrinogen in trauma patients and healthy controls were 230 mg/dL and 296 mg/dL, respectively (*P* = 0.031). Furthermore, traumatized patients had a significantly increased INR compared to the healthy controls (1.10 vs. 0.96, *P* < 0.0001). By contrast, there was no statistically significant difference in PTT between study groups, although there was a tendency toward slightly prolonged clotting time in controls (26.9 vs. 28.8 s, *P* = 0.116). Finally, serum calcium levels were significantly reduced following multiple injury (2.11 [n = 44] vs. 2.30 millimole per liter, *P* < 0.0001).

### Influence of trauma on clotting factor activity

**Fig. 2:**
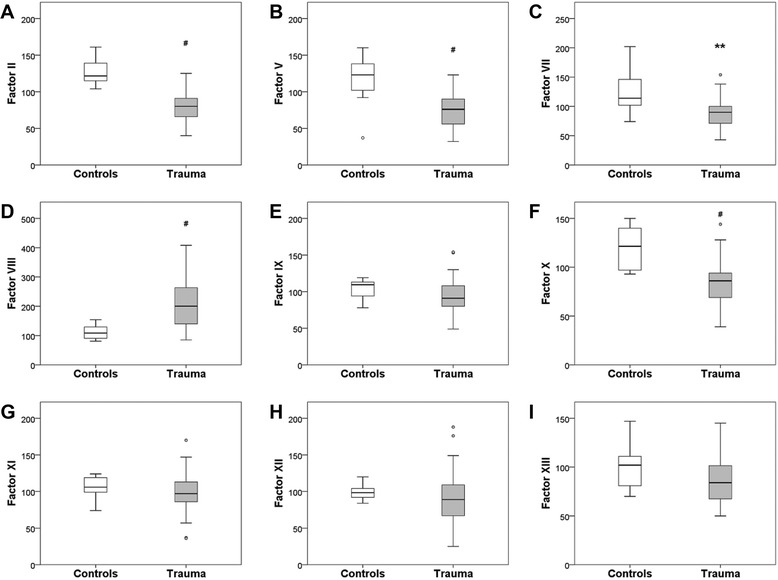
Changes in clotting factor activity after severe multiple trauma. The results are presented as boxplots, bottom and top of the box indicate the 25th and 75th percentile or interquartile range (IR). The horizontal bar within the box represents the median. Whiskers indicate spread (1.5 times IR). Outliers (1.5 to 3 times IR) are indicated by circles. The ordinate shows factor activities; (**A**) II, (**B**) V, (**C**) VII, (**D**) VIII, (**E**) IX, (**F**) X, (**G**) XI, (**H**) XII, (**I**) XIII. **, *P* < 0.01; ^#^, *P* < 0.0001

The median activities of all clotting factors were reduced after severe multiple trauma, with the exception of factor VIII, which was clearly increased. The results reached statistical significance for factors II (80 vs. 122 %, *P* < 0.0001), V (76 vs. 123 %, *P* < 0.0001), VII (90 vs. 114 %, *P* = 0.002), VIII (200 vs. 108 %, *P* < 0.0001) and X (86 vs. 122 %, *P* < 0.0001). Although not statistically significant, reductions in the median activity of factors IX (91 vs. 110 %, P = 0.141), XI (97 vs. 106 %, *P* = 0.270), XII (89 vs. 99 %, *P* = 0.295) and XIII (84 [n = 44] vs. 102 %, *P* = 0.09) were noted. Interestingly, factor VIII was the only variable, including routine coagulation tests, whose median was beyond the reference range of activity (70 – 150 % for this particular factor) set by the hospital laboratory. The results are presented in Fig. 2.

### Correlation of clotting factor activity and tests of coagulation

**Fig. 3:**
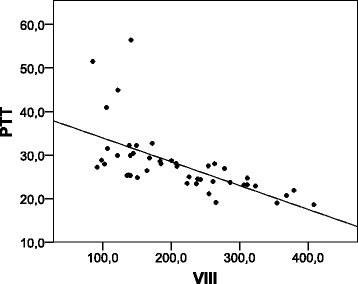
Graphical expression of the correlation between PTT and factor VIII. The dots represent pairs of PTT and factor VIII values. PTT, seconds; factor VIII, activity. The linear regression line is given; linear fit equation: factor VIII = −7 ∙ PTT + 404

For the patient group, Spearman’s Rank Correlation Coefficient *Rho* (*ρ*) for routine coagulation tests and clotting factor activity was calculated and is given in Table 3 with associated confidence intervals (CI). A strong (negative) correlation was observed between INR on arrival with serum fibrinogen and factors II, V, and VII (*P* < 0.0001). Correlation with the remaining factors remained moderate (*P* < 0.0001–0.005), with the exception of factor VIII. As shown in Fig. 3, PTT was strongly negatively correlated with factor VIII (*P* < 0.0001). Thus, PTT was the only variable with a relevant association with elevated factor VIII activity. PTT was also moderately correlated with factors II, V, IX, and XII (*P* < 0.0001). Levels of fibrinogen, also known as clotting factor I, were moderately correlated with levels of other factors, with the exception of factor XII (*P* < 0.0001–0.004), while calcium (factor IV) was barely associated with factors II, V, IX, XI, and XII (*P* < 0.01).

**Table 3:** Correlation between coagulation tests and clotting factor activity in patients

	F	II	Ca	V	VII	VIII	IX	X	XI	XII	XIII
INR	−0.72 (−0.85; −0.45)	−0.74 (−0.86; −0.53)	−0.46 (−0.72; −0.24)	−0.80 (−0.89; −0.58)	−0.75 (−0.88; −0.60)	−0.29 (−0.60; 0.05)	−0.54 (−0.78; −0.38)	−0.57 (−0.79; −0.38)	−0.44 (−0.70; −0.26)	−0.41 (−0.68; −0.18)	−0.42 (−0.63; −0.10)
PTT	−0.49 (−0.68; −0.13)	−0.63 (−0.76; −0.34)	−0.47 (−0.74; −0.20)	−0.65 (−0.79; −0.30)	−0.36 (−0.60; −0.06)	−0.77 (−0.88; −0.63)	−0.63 (−0.83; −0.45)	−0.51 (−0.74; −0.25)	−0.58 (−0.81; −0.41)	−0.58 (−0.79; −0.37)	−0.34 (−0.58; 0.00)
Fibrinogen	/	0.63 (0.36; 0.76)	0.54 (0.35; 0.73)	0.64 (0.39; 0.76)	0.47 (0.17; 0.67)	0.42 (0.13; 0.62)	0.54 (0.34; 0.76)	0.49 (0.22; 0.70)	0.44 (0.20; 0.68)	0.36 (0.11; 0.58)	0.53 (0.23; 0.74)
Calcium	0.54 (0.35; 0.73)	0.46 (0.22; 0.72)	/	0.40 (0.18; 0.66)	0.16 (−0.15; 0.43)	0.37 (0.08; 0.62)	0.50 (0.21; 0.72)	0.40 (0.10; 0.64)	0.46 (0.18; 0.67)	0.46 (0.21; 0.62)	0.38 (0.07; 0.64)

Data reported as Spearman’s Rank Correlation Coefficient, Rho (95 % CI)

*F* fibrinogen, *Ca* calcium

### Correlation of clotting factor activity and clinical data

No strong correlation between clotting factor activities and the clinical data of the patients was found. Apart from this, whereas the hemoglobin level at admission showed a moderate correlation with all clotting factors but factor VII (*P* < 0.0001–0.05), the remaining clinical parameters correlated only sparsely and mostly not significant with the various coagulation factors. *Rho* (*ρ*) for correlation between clinical data and clotting factor activity is given in Table 4 with associated CI.

**Table 4:** Correlation between clinical data and clotting factor activity in patients

	F	II	Ca	V	VII	VIII	IX	X	XI	XII	XIII
SBP prehospital	0.23 (−0.18; 0.69)	0.20 (−0.19; 0.71)	0.50 (−0.01; 0.80)	0.25 (−0.16; 0.68)	0.03 (−0.50; 0.50)	−0.10 (−0.46; 0.44)	0.16 (−0.27; 0.61)	0.15 (−0.22; 0.68)	0.05 (−0.44; 0.60)	0.11 (−0.23; 0.60)	0.03 (−0.35; 0.61)
SBP admission	0.20 (−0.46; 0.55)	0.31 (−0.19; 0.57)	0.21 (−0.22; 0.64)	0.36 (−0.18; 0.59)	0.11 (−0.45; 0.45)	0.14 (−0.32; 0.48)	−0.01 (−0.41; 0.49)	0.00 (−0.49; 0.42)	0.15 (−0.18; 0.71)	0.08 (−0.17; 0.63)	0.04 (−0.52; 0.49)
Lactate	−0.50 (−0.83; −0.22)	−0.44 (−0.73; 0.11)	−0.43 (−0.79; 0.08)	−0.43 (−0.74; 0.14)	−0.19 (−0.49; 0.42)	−0.19 (−0.66; 0.07)	−0.23 (−0.75; 0.29)	−0.15 (−0.56; 0.38)	−0.12 (−0.64; 0.35)	−0.08 (−0.54; 0.54)	−0.31 (−0.67; 0.08)
Base excess	0.23 (−0.08; 0.73)	0.38 (0.26; 0.77)	0.56 (0.12; 0.88)	0.57 (0.53; 0.86)	0.29 (−0.13; 0.81)	0.01 (−0.25; 0.53)	0.43 (0.46; 0.82)	0.12 (−0.11; 0.63)	0.25 (0.13; 0.76)	0.22 (0.04; 0.73)	−0.05 (−0.31; 0.54)
Temperature	0.18 (−0.25; 0.57)	0.46 (0.02; 0.85)	0.54 (0.08; 0.74)	0.30 (−0.18; 0.78)	0.12 (−0.54; 0.45)	−0.01 (−0.51; 0.41)	0.40 (−0.11; 0.71)	0.29 (−0.32; 0.69)	0.42 (−0.06; 0.79)	0.43 (0.00; 0.90)	0.05 (−0.25; 0.51)
Hemoglobin	0.46 (−0.06; 0.77)	0.52 (0.07; 0.74)	0.60 (0.30; 0.90)	0.45 (0.10; 0.73)	0.32 (−0.36; 0.66)	0.49 (−0.32; 0.53)	0.61 (−0.08; 0.65)	0.58 (−0.13; 0.69)	0.55 (−0.08; 0.71)	0.43 (−0.07; 0.63)	0.54 (−0.01; 0.81)
Thrombocytes	0.49 (−0.02; 0.79)	0.35 (−0.21; 0.68)	0.44 (0.03; 0.76)	0.18 (−0.29; 0.51)	0.17 (−0.34; 0.53)	0.33 (−0.46; 0.39)	0.31 (−0.37; 0.56)	0.36 (−0.26; 0.58)	0.39 (−0.17; 0.71)	0.29 (−0.23; 0.60)	0.40 (0.16; 0.77)

Data reported as Spearman’s Rank Correlation Coefficient, Rho (95 % CI)

*F* fibrinogen, *Ca* calcium, *SBP* systolic blood pressure

## Discussion

The statistical analysis did not reveal statistically significant differences in the baseline demographics of patients and healthy controls. We assume that both groups were comparable and changes in clotting factor activity after severe trauma could be analyzed. A mean ISS of 31 points and an overall mortality of 27 % in patients also implied relevant tissue traumatization. The predominance of blunt injury reflects the medical situation in Germany and has been described previously [7]. However, this might have implications for the generalizability of these findings to different patient populations as they might differ in the incidence of penetrating trauma [22]. Because (isolated) penetrating trauma leads to uncontrolled hemorrhage rather than exorbitant tissue damage and the pathophysiology of the observed changes may depend on the trauma mechanism, divergent findings could be obtainable. One aim of this study was to test for clotting factor impairments as early as possible following trauma to exclude the influence of treatment. In Germany, prehospital emergency physicians are involved in patient care, and thus a potential bias related to individualized patient care prior to admission cannot be fully excluded. Hence, during the enrollment period no general modification of routine prehospital trauma life support was implemented in the study area. In-house protocols for the initial care of polytraumatized patients were also not modified. Furthermore, routine resuscitation room algorithms in our institution provide for blood sampling at the earliest possible time point. In a multidisciplinary strategy, blood is regularly drawn immediately after patient arrival and usually occurs prior to the placement of central venous lines. This strongly reduces the potential interference of the different tests with relevant fluid resuscitation. Laboratory analysis itself may be affected by the variable degradation timeline of clotting factors. Again, this cannot be fully excluded because several clotting factors showed different half-lives [23]. However, because blood specimens were immediately transported to the laboratory after withdrawal and, if necessary, promptly deep frozen, this is unlikely.

As all primarily admitted patients irrespective of any kind of predefined coagulopathic status were included and results were compared with a reference group, this study differs significantly from the existing ones [19, 20]. The findings of this study demonstrate that, with the exception of factor VIII, clotting factor activities were generally reduced in the early time period following injury. Amongst others, serum levels of fibrinogen and calcium were reduced significantly, a finding that has been described by others [6, 24]. However, our results do not support studies according to which fibrinogen was the earliest and predominantly reduced factor after traumatic blood loss [25, 26]. Consistent with the study of Rizoli et al., we found the activity of factor V the most deficient [19]. This predominance of a factor V deficiency is a strong indicator of an activation of the protein c pathway, which in turn seems to play a key role in the onset of acute traumatic coagulopathy as proposed by Brohi et al. [10, 11, 19]. Hence, the rather low correlation of clinical parameters of tissue hypoperfusion (SBP, lactate, BE) with clotting factor activities is somewhat contrary to the concept of shock induced coagulopathy also supposed by Brohi et al. [10, 11]. In general, the median activities did not fall below the given reference range. This reflects the rather minor rate of only twenty percent of coagulopathic patients according to the inclusion criteria in the study of Rizoli et al. As the authors stated, the threshold of 30 % of activity is somewhat arbitrary and the clinical importance of a less dramatic but simultaneous decline in the activity of multiple clotting factors is unclear. Indeed, in our study the median activities of clotting factors in the normal cohort frequently exceeded the “standard” activity of 100 % and therefore the absolute reduction is even higher. Additionally, those reference ranges were originally established to assess single factor deficiencies and may not be appropriate for trauma patients. Therefore, the imminent reduction in almost all clotting factor activities should not be underestimated as it might act as a potential contributor to trauma-associated coagulopathy. In conclusion, these findings should be considered when treating hemorrhagic patients after severe multiple trauma, as higher ratios of clotting factors might be needed to restore normal coagulation function. Factor concentrates such as prothrombin complex concentrate contain clotting factors in high concentrations and might prove beneficial for treating the deficiencies noted in our study. With respect to the increased activity of factor VIII, our results are consistent with the studies of Cohen and Jansen et al. [20, 27]. The latter is based on a subgroup analysis from the work of Rizoli et al. and reported a factor VIII activity level beyond the upper limit of the range in 72 % of all patients. Indeed, elevation of factor VIII activity seems to be the most robust effect of severe multiple trauma in terms of clotting factor activities. A possible explanation is the known role of factor VIII as an acute phase protein [28]. However, because our study design allowed for blood withdrawal quite soon after injury, this might not fully explain the detected levels of factor VIII at such an early time point. Further explanations involve a direct liberation of factor VIII from injured vessels and damaged tissues by unknown mechanisms or, alternatively, active secretion. This might be reasonable, as factor VIII is known to be produced by a wide range of cells [29, 30]. However, the physiological relevance of elevated factor VIII activity after severe trauma remains unclear.

Prothrombin Time (PT) and PTT have major limitations in the diagnosis of trauma induced coagulopathy as they are time-consuming and lack the desirable sensitivity at the critical time of admission. In addition, it is unclear to what extent possible derangements of the underlying coagulation system are reflected by PT and PTT [31]. This has resulted in the emergence of viscoelastic tests (rotational thromboelastometry [ROTEM] or thromboelastography) as a point-of-care diagnostic procedure for detecting acute coagulopathy after trauma [32]. In experimental hypothermia and hemorrhage, ROTEM showed superiority over PT and PTT in predicting coagulation disorders and mortality [33, 34]. In this study, INR (reflecting PT) was significantly elevated whereas differences in PTT were negligible. Indeed, an abnormal PTT is known to occur more infrequently than changes in PT [5]. The strongly elevated factor VIII levels found in this study offer a possible explanation for this phenomenon. As indicated by a strong negative correlation, high plasma levels of factor VIII may “discredit” the measurement of PTT, potentially leading to a reduced (quasi normal) clot formation time *ex vivo*. In this context, PTT would be useless in an attempt to diagnose traumatic coagulopathy. If viscoelastic tests are not available, INR might be helpful as it highly correlated with reduced levels of fibrinogen and the activities of factors II, V, and VII. This finding is reasonable because INR reflects PT, which was designed to test these factors (formerly called the “extrinsic pathway”). Under these circumstances, INR might be used despite its known limitations to trigger coagulation therapy, e.g., by use of fibrinogen concentrate and PCC. Nevertheless, future studies are desirable to elucidate the potential of viscoelastic tests to predict underlying clotting factor deficiencies in trauma induced coagulopathy.

## Conclusions

This prospective study compared clotting factor activities in patients during the early period following severe multiple injury with a normal cohort. With the exception of factor VIII, activities of clotting factors are moderately reduced. This should be considered in the initial treatment after severe multiple trauma. In the concept of a calculated coagulation therapy, this could demand for the use of factor concentrates with higher ratios of clotting factors. Finally, although the physiological importance of elevated factor VIII activity after severe trauma remains unclear, a possible interference with PTT measurement *ex vivo* has to be considered.

## Abbreviations

CI: Confidence interval
INR: International normalized ratio
IR: Interquartile range
ISS: Injury severity score
PT: Prothrombin time
PTT: Partial thromboplastin time
ROTEM: Rotational thromboelastometry
SBP: Systolic blood pressure
SD: Standard deviation
SHP: Standard human plasma
